# Home-Telemonitoring Lung Cancer Intervention in Appalachia: A Pilot Study

**Published:** 2016-05-25

**Authors:** YJ Chen, GL Narsavage, KD Frick, TM Petitte

**Affiliations:** 1 Kent State University, Kent, OH, USA; 2 Robert C. Byrd Health Sciences Center, West Virginia University, Morgantown, USA; 3 Carey Business School, Baltimore, USA; 4 West Virginia University, Morgantown, USA

**Keywords:** Home-Telemonitoring, Lung Cancer, Appalachia, Self-Management, Randomized Clinical Trial

## Abstract

Benefits of home-telemonitoring for rural dwelling cancer patients are largely unknown. This study examined the effectiveness of home-telemonitoring surveillance with nurse coaching for self-management to improve lung cancer outcomes in mountainous Appalachia where health care access/ service is limited. This randomized clinical trial pilot study compared patient outcomes for telemonitoring versus routine care. A convenience sample (*N* = 47) was enrolled/ randomized (Telemonitored: 26/ Control: 21) from a university hospital and cancer center. Physiologic parameters and symptoms were collected in the telemonitored group for two weeks; all participants were studied for 60 days after the index treatment/ discharge. The telemonitored group showed greater improvement for both functional status (*Wald X^2^* = 3.78, *p* = .05) and quality of life (QOL) (*Wald X^2^* = 7.25, *p* = .007) from baseline to 60 days post-discharge. Compared to controls, telemonitored patients survived longer; had more scheduled medical visits (96% vs. 75%); made more unplanned calls to doctors/ nurses (32% vs. 30% & 64% vs. 50%); had fewer rehospitalizations (28% vs. 40%); and had more ER utilization (36% vs. 30%). The telemonitored group had relative improvements for health utility (.09 on a scale where 0 = death/ 1= perfect health) and QOL (15 on 0–100 VAS). Differences in health care utilization and cost were not significantly different (*p* > .05), likely due to the sample size. Telemonitoring group satisfaction with care was high and recommended by patients and caregivers. Results suggest that it is possible to improve patient outcomes with home-telemonitoring for self-management in rural areas. Short-term, telemonitoring-based coaching is feasible and offers a promising option to develop patient self-management knowledge and skills.

## Introduction

In 2016 lung cancer caused 26–27% of all cancer deaths in the United States (U.S.), consistently more than any other cancer for both men and women for the past two decades [1, 2]. Lung cancer incidence and mortality in West Virginia (WV) exceeds U.S. averages and ranks as the most frequent cancer diagnosis in the state [2–4]. Appalachian residents in WV are geographically isolated, generally of low economic status, tend to delay or inadequately use medical care, and have significant health disparities compared with non-Appalachian residents in less rural areas [5, 6].

High rehospitalization rates, low functional status, and poor treatment outcomes among lung cancer patients are attributed to environmental factors that result in a large proportion of patients being diagnosed when they are older and at later stages of the disease [3, 6]. Patients with lung cancer and their family caregivers frequently experience unmet needs and lack supportive care. Learning to deal with complex cancer symptoms during their disease trajectory is a challenge. There is a critical need for low-cost interventions to assist patients with self-management in their residential community. Applying appropriate home-health technology can be key to helping rural dwelling patients and caregivers to develop self-management skills needed to live with their diseases [7]. The literature supports several benefits identified by patients and family caregivers using home monitoring devices: early detection of physical changes, problem-solving self-management using available telemonitor data, and improved communication between health care providers and patients [7–9].

Telehealth/telecare has been evolving as a helpful influence applied to current health care systems, policies and practices [10–13]. Particularly for the chronically ill, older patient population, telehealth has demonstrated the potential to improve clinical outcomes [14–16]. In recent systematic reviews [8, 13, 17], home telehealth benefits for chronic diseases were summarized as improving health care communication, patient education and related health outcomes (e.g., better quality of care, quality of life, and social support), cost-effectiveness, health-knowledge, self-care and health-management. However, telehealth outcomes have been inconclusive due to a lack of robust research designs as well and broad descriptions of approaches being applied [8, 12]. In addition, limited evidence is reported for telehealth in oncology, especially studies focused on the first two weeks following hospital/initial cancer therapy discharge, before scheduled follow-up visits – a critical symptom-management time. There were no identified studies using home-telemonitor data to inform nurse-coaches and educate adults with lung cancer.

In this pilot study that was preceded by a feasibility study [18], the objective was to assess the design, implementation and challenges of conducting a randomized clinical trial (RCT) of home-telemonitoring surveillance to develop self-management skills that could improve outcomes for Appalachian adults with lung cancer. The intervention included nurse coaching to help patients proactively manage their multifaceted conditions. The specific aims were to (1) describe changes in physiological measurements and subjective symptoms over 14 days following discharge in the telemonitored group; (2) identify differences in functional status, quality of life (QOL), and satisfaction with home telemonitor-based education for self-management; and (3) analyze differences in nurse/physician contact, health care utilization, and costs between groups. Patients with lung cancer were hypothesized to improve self-reporting signs/symptoms to their clinicians and decrease use of costly health care resources over 60 days after receiving home-telemonitoring surveillance and post-discharge nurse coaching. We anticipated telemonitors would provide early evidence of disease-related changes that could be recognized by patients and relayed to clinicians before patients reached a critical stage.

### Conceptual Framework

The study’s conceptual framework was derived from the Chronic Care Model (CCM) for evaluating “patient-centered” health outcomes, functional status, QOL, satisfaction with care, and decreased use of health care services [19]. Health outcomes could be impacted by interactions among nurses, patients, clinicians, and patient’s self-management. The model’s “community resource system” intervention strategy was a program of support that involved nurse-guided education and coaching (decision support). Coaching helped patients acquire knowledge needed to recognize critical changes in physical signs and symptoms and to adopt associated behaviors to “adjust their roles” (to contact clinicians) for optimal function, control of their symptoms, and improvement of their well-being. Patient factors, including age, disease severity (cancer stage), pain, dyspnea, and comorbidity were collected from the hospital/cancer center’s electronic medical records. The CCM provided a structured framework to guide the study in bridging the community, hospital, and health system to improve patient-centered outcomes of cancer care.

## Materials and Methods

This study examined the effectiveness of a home telemonitoring system to aid patients discharged with lung cancer in understanding how and when to contact clinicians and avoid rehospitalization. Our approach addressed existing challenges of remote/rural care using telemonitor-identified real-time physiologic and symptom data.

### Study Design and Participants

**Figure 1:**
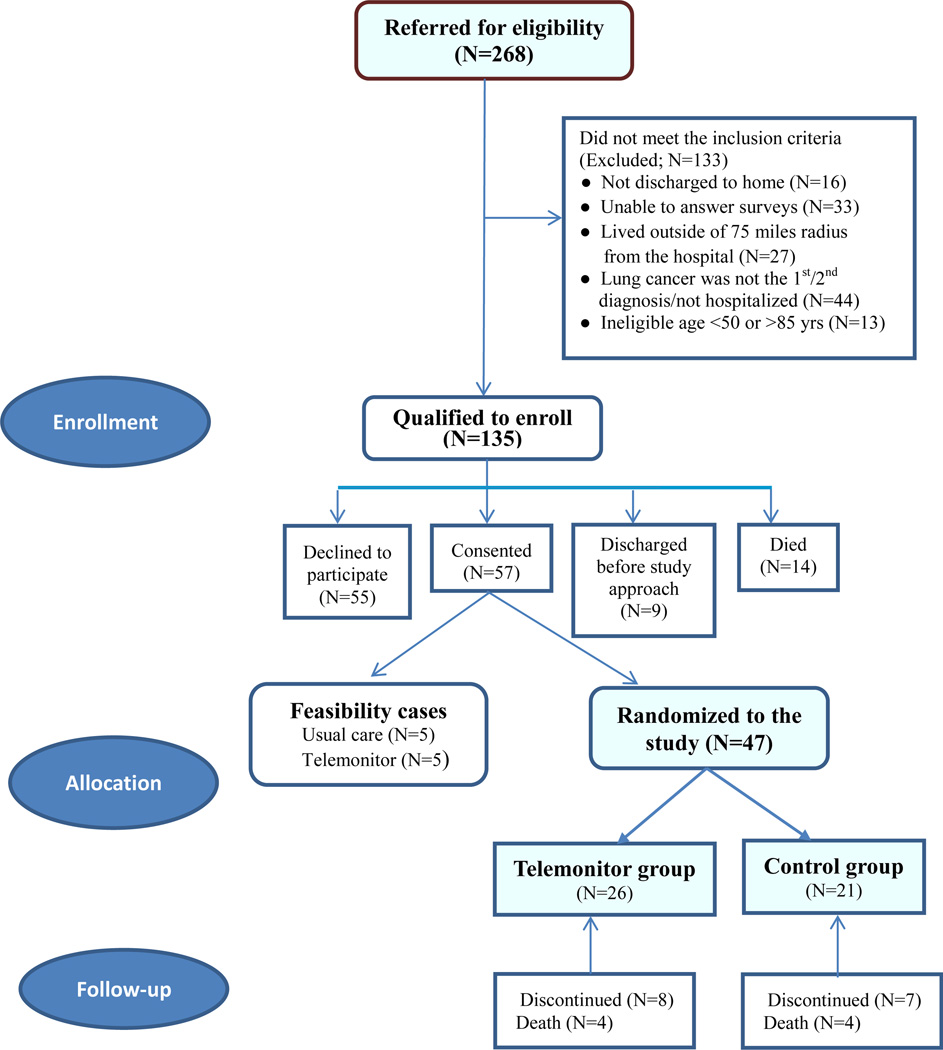
Study Patient Enrollment Flow Chart

This pilot randomized clinical trial (RCT) compared telemonitored care and usual post-discharge care for lung cancer patients in Appalachia. In 2012, a total of 268 potential patients from a teaching hospital/oncology care center in north-central WV were referred and then screened. The majority (> 90%) of the study patients resided in non-metropolitan areas defined as rural Appalachian communities outside the city of Morgantown, including areas in WV, Pennsylvania, and Ohio. Inclusion criteria were (1) hospitalized due to lung cancer with discharge to home, or having active cancer treatment for lung cancer as a primary or secondary diagnosis; (2) between 45 and 90 years old; (3) cognitively alert; and (4) able to speak English. Patients were excluded if they were discharged with hospice care or lived outside a 75-mile radius from the study hospital. As reflected in Figure 1, approximately 50 percent of the referrals were disqualified/excluded, and 135 qualified for the study. Of the eligible referrals, 55 patients (40.7%) refused to participate for personal/ family reasons; nine were discharged prior to consent; 14 died in hospital; and 57 consented. The study initially studied 10 non-randomized feasibility patients whose data were published previously [18]. The 47 remaining participants were randomly assigned to either the usual care control or telemonitor intervention group and included in the final analyses (usual care controls: 21; telemonitored: 26). By the end of the study, eight patients had died, and 15 others withdrew from the study.

### Telemonitoring Intervention

**Figure 2:**
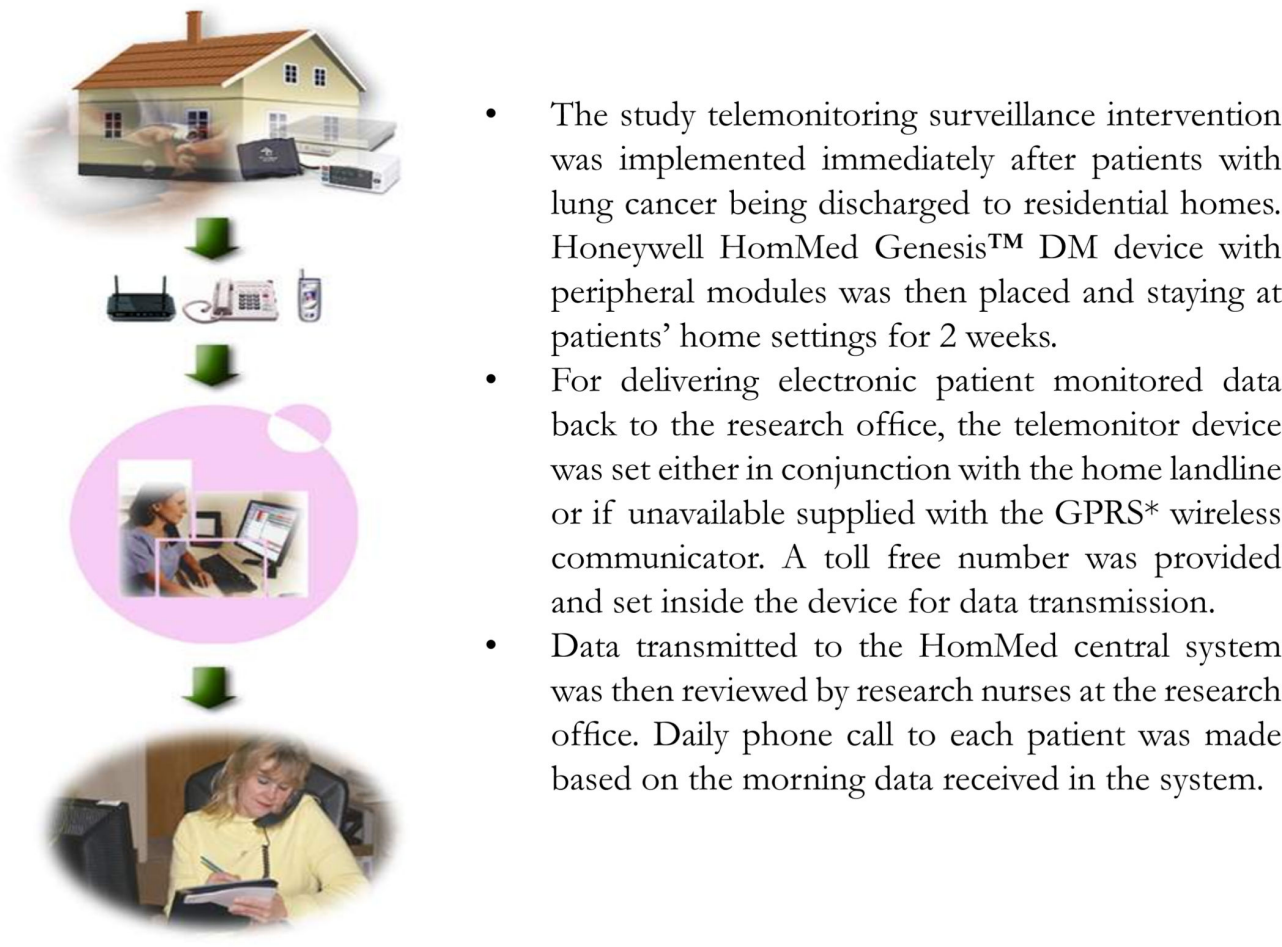
Study Protocol Telemonitoring Surveillance Intervention *General Packet Radio System (GPRS)

The study intervention used a wireless, in-home telemonitoring system (Honeywell HomMed Genesis™ DM) and patient-centered phone coaching by nurses in addition to post-discharge usual care (see Figure 2). Objectives of the telemonitoring surveillance were to use nurse coaching based on the data to help patients (1) lengthen periods out of hospital; (2) support self-management of disease-related conditions (e.g. lung cancer and comorbidity symptoms, medications) using home oxygen, medications, and/or problem-solving; (3) reduce unplanned hospitalizations and emergency room (ER) visits; (4) decrease overall health care costs; and (5) improve patient functional status and quality of life.

An eight-hour equipment training course was delivered to all research team members by Honeywell HomMed, Inc. Clinical assessment modules and symptom questions for the population under study were then customized and built into each device as well as into the LifeStream™ software platform. The surveillance design included placement of a telemonitor in each intervention patient’s home post-discharge from hospital or cancer clinic, to be used with nurse coaching for two weeks before scheduled follow-up clinician visits. Patients’ physiologic parameters and symptoms were measured on a daily basis for 14 days. Intervention patients were scheduled to transmit telemonitored data to the study office each morning to ensure consistency of data collection. In addition to their morning monitoring, they were free to use the device anytime if they felt a need for immediate measurement. Research nurses read the data received daily through the LifeStream™ platform and called patients to interpret results using a questioning/coaching technique as needed. Telemonitored data alerted nurses to changes in patients’ daily conditions. Nurses explained changes in physiological signs and symptoms and, based on motivational interview training, coached patients on how to problem solve and when to contact their oncology clinicians. The daily monitoring protocol design also provided early evidence of disease-associated changes. Thus, patients were coached to develop self-management skills and relay recognized changes to their clinician before they became critical. More detail on the telemonitoring process itself is described in the previous feasibility study publication [18].

### Data Collection

A total of five data collection time-points were used for both study groups: Time 1 (T1)– a hospital/clinic visit for enrollment; Time 2 (T2)– a home visit within 48–72 hours of discharge; Time 3 (T3)– a home visit at 14 days; Time 4 (T4)– a phone call follow-up at 30-days; and Time 5 (T5)– a home visit at 60 days post-discharge. The telemonitored group received 14 days of home-telemonitoring and nurse coaching following the hospital/ clinic discharge. The control group had usual care as ordered in their discharge plan which might include home health care services and office/ clinic scheduled visits, plus the data-collection home visits/ phone calls for collecting study data. During the study time periods, data collection was continuously monitored. Inter-rater reliability > 90% between data collectors was maintained throughout the study. Total one-year health care costs were obtained through hospital/outpatient administrative systems for each patient participant.

### Measurement of Variables

Independent variables were study groups, patient demographics, and physiologic baseline measures. Physiologic measurements were collected to detect changes in an individual’s day-to-day condition of chronic and progressive symptoms that could impact activities of daily living. Daily telemonitored data for intervention participants included objective parameters (temperature, pulse rate, blood pressure, weight, and SpO_2_) and 10 subjective symptom assessments as shortness of breath, cough, tiredness, limited activity, nausea/ vomiting, pain, chest pain, standing/ walking, appetite, and anxiety in comparisons of today with yesterday. Telemonitor variables were also collected by research nurses for both groups at all study data time points, except T4.

Patient outcomes were measured by functional status (the short-form Pulmonary Functional Status Scale; PFSS-11), quality of life (the WHO-5 Well-Being Index; WHO-5), satisfaction with telemonitor care, and utilization of health care resources. The PFSS-11 assesses daily activities, social and psychological functional status [20]. The WHO-5 was developed to indicate an individual’s well-being and reflect depressive symptoms [21, 22]. Its components include mental status, social relationships, environment, and self-perceived health status. All instruments had established high validity and reliability [20, 21]. Patient/ family satisfaction with telecare was measured by an eight-item ordinal scale of perceived satisfaction from strongly disagree (dissatisfied) “0” to strongly agree (satisfied) “4” at T3 –T5. A cooperative agreement with Honeywell allowed use of their telemonitor satisfaction survey for this study (calculated internal reliability Cronbach’s alpha of .86).

An investigator-developed outcome form, “health care resource utilization,” included data on frequency of clinician/ nurse contacts, physician office visits (scheduled or unscheduled), home care nurse visits (scheduled or unscheduled), rehospitalization, and ER visits. Health utility measures (EQ-5D index and one-year direct medical cost) were calculated to evaluate potential cost-effectiveness in comparison with usual care. The EQ-5D-3L (EuroQOL five dimensions with three level version) is a generic measure of health outcomes as well as health utility [23–26]. The utility score is widely used in economic analysis as the outcome for cost-effectiveness [25, 26]. The score is an input to quality adjusted life years (QALYs) and is calculated as a combination of health attributes and quality of life (1–100 rating scale of the EQ-VAS). The scoring algorithm of health attributes is generated as a single index value ranging from 0–1 (zero (0) = death and 1 = perfect health) [23, 24]. The extent of direct health care service costs for patients throughout the entire study, 60 days, was approximated by analyzing one-year of hospital services in three sections: pre-study (six months), during the study (two months), and post-study (four months). Calculations used the Centers for Medicare and Medicaid Services (CMS) national mean payment per day for type of service for 2011 (ER $1,354, observation $1,400, inpatient $2,420) as well as cost by type of care.

### Data Analysis

The Statistical Package for Social Sciences (SPSS) version 22.0 was used for data entry and analyses, with additional cost analyses completed using STATA standard package (version 13.1; http://www.stata.com). Descriptive analyses were performed on baseline and clinical characteristics of the study groups, and two-sample t-test and chi-square were used to examine group differences. Telemonitored data were compared by means/percentages, and these parameter changes were observed over time for patterns by horizontal graphs. The intention-to-treat approach was applied including all randomly assigned participants in the final study analyses, regardless of missing outcome data, by a generalized estimating equations (GEE) method. The GEE was performed for longitudinal data analyses of correlated response estimates with missing values [27]. In this study, the GEE method was used to examine the potential effect of home-telemonitoring on repeated patient outcomes at five time points across the two months of the study. To analyze the cost-effectiveness outcome, differences in health utility measures were evaluated by grouping Mann-Whitney tests at study time-points compared to baseline. We were unable to estimate cost-effectiveness ratios as we realized that the small group would make it challenging to calculate meaningful cost-effectiveness ratios. Analyses comparing costs before, during, and after the interventions were conducted using STATA. In addition, an exploratory analysis was conducted based on participants with compliance versus non-compliance to participation/ data reporting. Participants defined as compliant completed ≥ 80% of the study/ data reports. Differences and trends in health care utilization were distinguished by compliance versus non-compliance within and between study groups. The statistical significance level was set at *p* < .05.

## Results

### Demographic and Clinical Profiles

Demographic and clinical characteristics for study participants are listed in Table 1. The majority of participants were Caucasians (98%) and had been newly diagnosed (< one year; 77.8%) with NSCLC (91.5%) at stage IIIB–IV, including those with metastasized lesions (66%). Groups were thus comparable in cancer staging. A majority of participants were older (mean age: 63±9.9 years), male (55%), married (68.1%), with high school or less education (76.6%), previous/current smokers (95.7%), overweight/obese (61.7%), and covered under Medicare (51%) with frequent prior hospitalizations (77%). Between groups, none of the baseline characteristics were statistically different (*p* > .05) except for previous hospitalization - telemonitored patients had more hospitalizations in the previous year than the controls (88.5% vs. 61.9%; *X^2^* = 4.57, *p* = .03).

### Telemonitored Changes over 14 Days Post-discharge

In the telemonitored group, physiological measurements fluctuated but revealed no significant patterns over time following discharge. Average values with ranges of physiological measurements at baseline versus the 60-day end of study are shown in Table 2. Patterns of subjective symptoms for the telemonitored patients did show trends for decreased tiredness, nausea/ vomiting, pain, chest pain, difficulty in standing and walking, and anxiety, as well as improved appetite over the two weeks post-hospitalization. At baseline, 19% of telemonitored patients were experiencing dyspnea, and at 14 days, 25% reported increased dyspnea–findings consistent with their greater morbidity based on previous hospitalizations. There was also a slight although statistically insignificant increase (48% vs. 50%) in activity limitation and coughing (19% vs. 25%) from baseline to 14 days after hospital/clinic visits.

### Differences of Patient Outcome Measures

**Figure 3:**
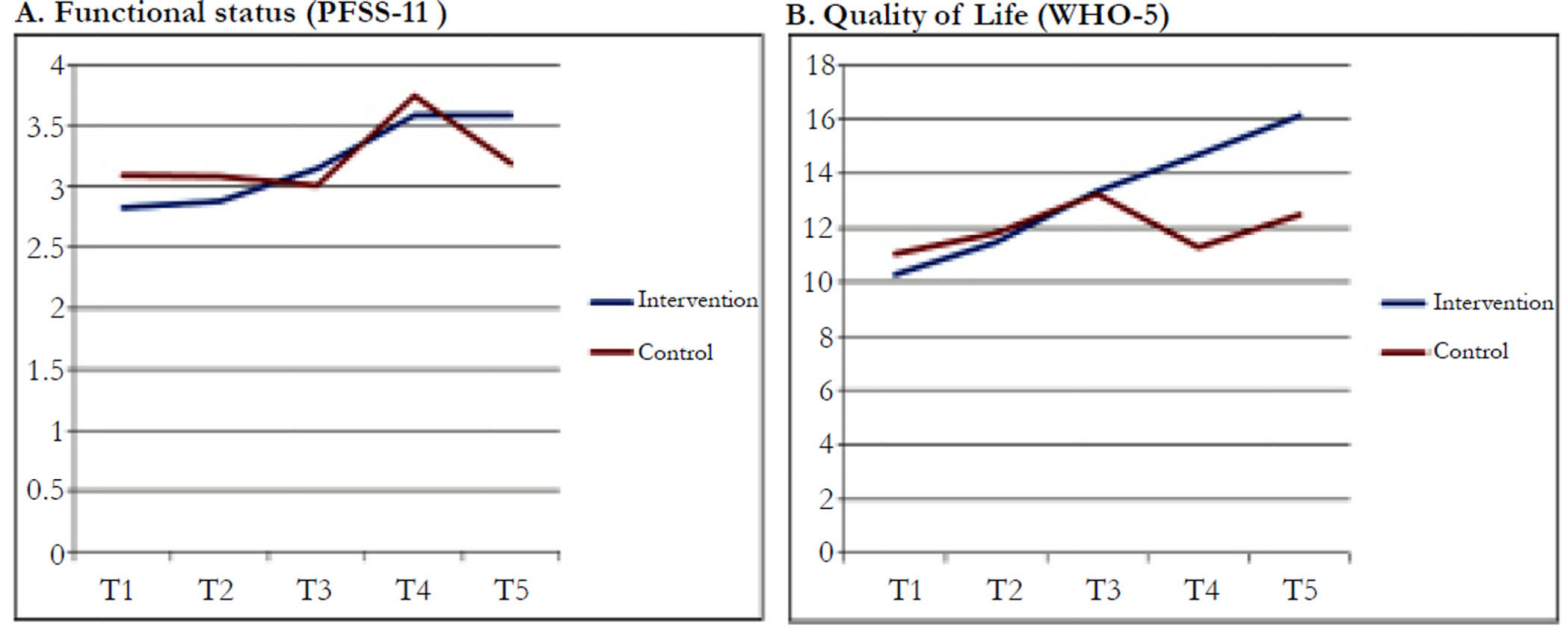
Differences in Functional Status and Quality of Life (QOL) Over Time Notes. T1: baseline hospital/clinic visit before or at discharge; T2: within 48–72 hours post discharge/visit; T3: 14 days post discharge/visit; T4: 30-days post discharge/visit; T5: 60-days post discharge/visit. PFSS-11 Mean score of 11 items: 0 (not able to do activity) to 5 (no difficulty); possible range 0–5. WHO-5 Sum score of the 5 answers to feelings (e.g., cheerful) on a likert scale from 0 (none of the time) to 5 (all of the time); possible range 0 to 25. 0 represents worst possible quality of life and 25 represents best possible quality of life.

The PFSS-11 scores documented that functional status improved in the telemonitored group over time (Average score of T1 vs. T5: 2.8 vs. 3.6 in telemonitored group & 3.1 vs. 3.2 in control; *Wald X^2^* = 3.78, *p* = .05, 95% *CI* = .001– .194). Quality of life scores improved from baseline to 60 days after hospital/clinic discharge in both groups (WHO-5 mean scores, telemonitored: 10.3 vs. 16.1; control: 11.1 vs. 12.5; *Wald X^2^* = 7.25, *p* = .007, 95% *CI*= .259–1.647). The telemonitored group had consistency in direction for both functional status and QOL compared to the control group over time; whereas, the control group had irregular variability in levels of patient outcomes with narrower improvements compared to the telemonitored group. Nevertheless, between groups in Figure 3, telemonitoring versus control had no significant difference observed on outcomes in the GEE results (*p* > .05).

Participants’ satisfaction, reflected by available data (*N* ≤ 10), indicated that study patients and family members would like to use telemonitoring in the future and would recommend telemonitoring care to others (mean score = 3.3–3.8, *SD* = .3–1.2). They reported that using the telemonitor helped them feel more involved in their care, gain a better understanding of their condition, and manage their health while providing a sense of security and peace of mind.

### Differences in Health Care Utilization

**Figure 4.1:**
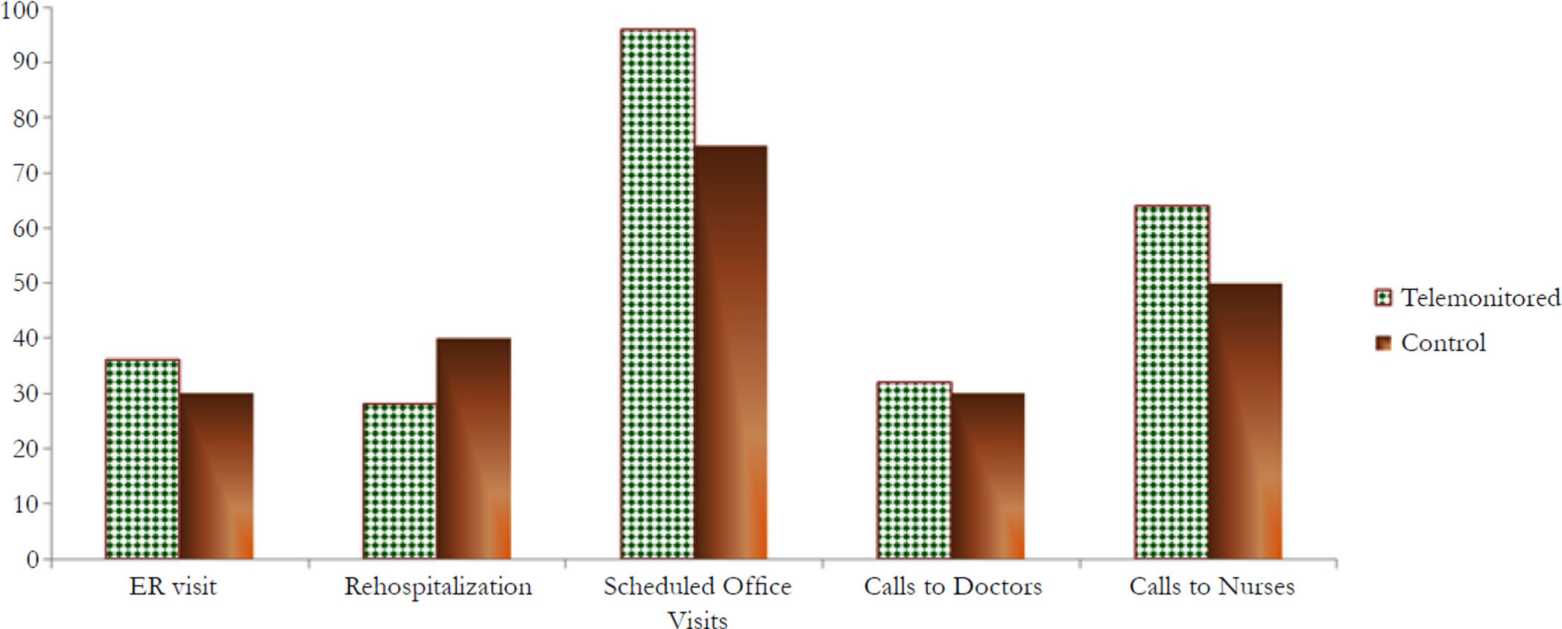
Comparison of Health Care Utilization (%) Between Telemonitored and Control Groups (*N*=47) During The Study Time Periods Note: The % of patients who used health care (ER, Hospital, Office visits, Calls to doctors and calls to nurses) are calculated as number of patients for whom an event was recorded divided by the possible number of patients who could have experienced the events. Overall, use of health care services were more likely to occur in telemonitored participants than controls, including ER services, scheduled medical office visits, and unplanned calls to doctors and nurses. Noticeably, more rehospitalizations were shown in control group compared to telemonitored patients (40% vs. 28%).

There were no statistical differences and patterns in patient outcomes over time with regard to health care utilization and cost between groups. In comparison with the control group, telemonitored patients had more scheduled medical visits (96% vs. 75%); made more unplanned calls to doctors and nurses (32% vs. 30% & 64% vs. 50%, respectively), had fewer rehospitalizations (28% vs. 40%), and had slightly higher use of ER services (36% vs. 30%; see Figure 4.1). The fact that telemonitored patients survived longer could explain the higher use of services.

Comparing the changes from baseline to the end of the study, health utility data showed relative improvements in the telemonitored group as large as .09 on the EQ-5D scale (non-significant difference between the index scores: .68 vs .77, *p* = .63) and a substantial 15 points on a 0–100 VAS (61 vs. 76, *p* = .04). For health care costs during the three time periods examined over a year, the telemonitored group had higher in-hospital costs leading up to entry into the study and during the index admission period (two months) compared to controls. However, in the post-intervention period (four months), the costs were lower, on average, for telemonitored patients who also survived longer. Nevertheless, looking at the costs over the entire study period with the assumption that all patients with “no data” had died and had zero costs, we found that the overall costs were even lower in the control group (see Table 3). The data indicated that longer survival overall costs more than shorter survival as seen in the control group.

### Exploratory Analysis by Study Compliance versus Non-Compliance

**Figure 4.2:**
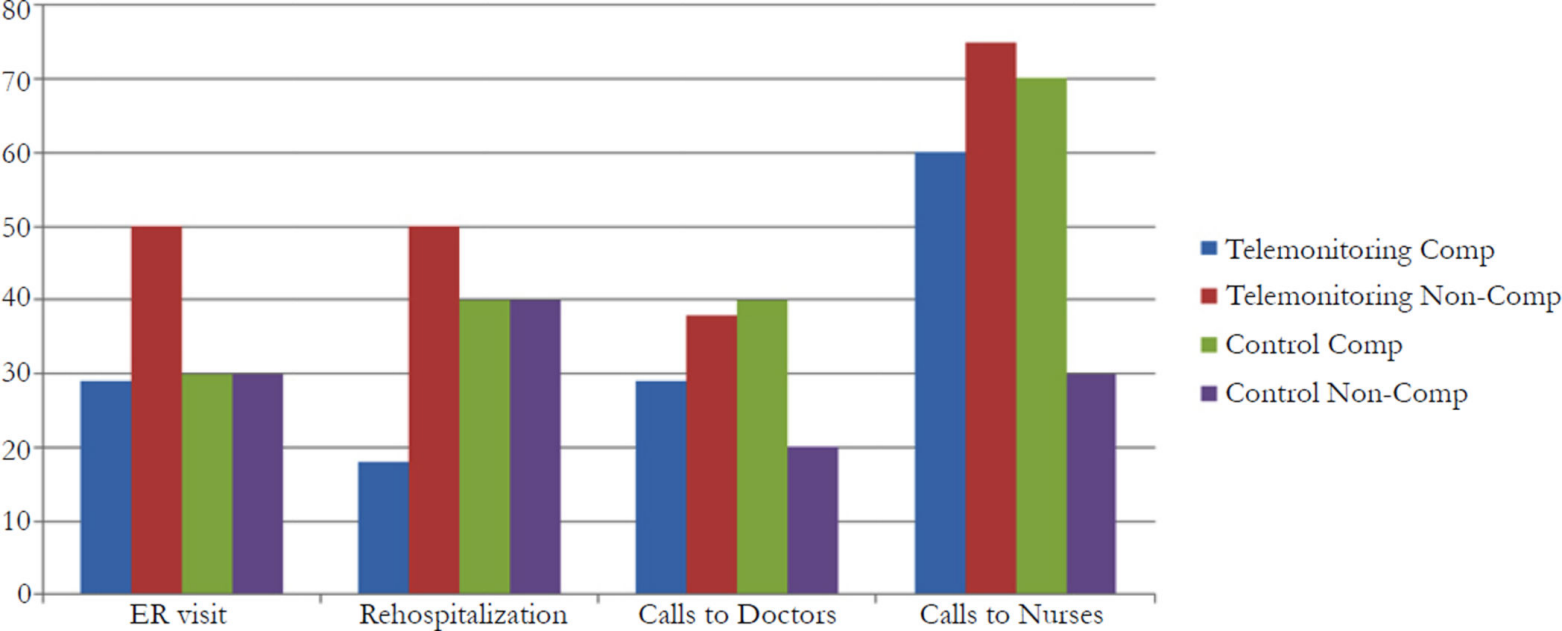
Comparison of Health Care Utilization Outcomes (%) Between Telemonitored and Control Groups With and Without Compliance to Study Data Reporting Note: The % of patients who used health care (ER, Hospital, Office visits, Calls to doctors and calls to nurses) are grouped by study compliance for both intervention or control groups calculated as number of patients for whom an event was recorded divided by the possible number of patients who could have experienced the events. Generally, the compliant patients made more scheduled and unscheduled calls to doctors and nurses than those who were non-compliant. As a consequence, the telemonitored compliant group revealed a positive outcome in fewer acute care visits compared to the other three subgroups (ER visit: 29% < 30–50%; rehospitalization: 18% < 40–50%). In contrast, non-compliant and control patients used more acute care services than those who were compliant with the study telemonitoring protocol.

Approximately 60% of the sample ware compliant versus 40% who were non-compliant with data reporting (participation) in the study. Patients in the telemonitored group were more likely to be compliant with study protocol than those in the “usual care” group (69% vs. 48%).Within 60 days of post-hospital/outpatient visits, non-compliant patients used more acute care services than those who were compliant (subtotal frequency: ER visit: 10 > 8; rehospitalization: 13 > 9). And, as anticipated based on nurse coaching and self-management, the compliant patients made more calls to doctors and nurses than those who were noncompliant (doctor calls: 14 > 6; nurse calls: 53 >16; see Figure 4.2). Calls were considered a desirable outcome versus use of emergent care. Although not statistically significant, of clinical significance was that the telemonitored compliant group had the least number of ER visits and rehospitalizations compared to the other three subgroups: telemonitored non-compliant, control compliant, and control non-compliant (ER visit: 29% < 30–50%; rehospitalization: 18% < 40–50%, respectively; *p* > .05).

## Discussion

This pilot study aimed to assess the design, implementation and challenges of conducting a randomized clinical trial (RCT) of home-telemonitoring surveillance to develop self-management skills that could improve outcomes for adults with lung cancer who live in remote/rural Appalachia. The challenge of enrollment and retention inhibited finding statistically significant differences in outcomes. Nevertheless, clinical significance supports future study. Study results indicated positive patient outcomes and decreased use of acute care services among compliant telemonitored patients compared to the group receiving routine care. Outcome improvements in this study included enhanced functional status and QOL, positive satisfaction with home-telemonitoring for self-management and lower acute-care utilization, as well as lower follow-up costs four months post-study compared to the six-month time period prior to the study.

Patient physiological and symptom measurements fluctuated with no significant difference throughout the two-week daily home-telemonitoring. Although the intervention group’s subjective symptoms decreased at the end of telemonitoring, physical conditions such as dyspnea, coughing and activity limitation seemed to worsen over the following six weeks, likely due to the nature of the cancer disease prognosis itself. Perhaps of clinical significance, this deterioration was not statistically significant. This finding was congruent with the results of a recent RCT study with unsuccessful management to ease lung cancer symptom burden after undergoing weekly telephone monitoring for three months [28]. However, in other studies telemonitoring was helpful for improving cancer symptom reporting to clinicians using personal computer/tablets as well as increasing cancer treatment adherence and patients’ capability of self-management [29, 30]. Although telecare could contribute to a degree of cancer symptom control, the effects are not strong enough to overcome the morbidity of the disease itself in terms of patient and clinical outcomes.

Telemonitored participants, particularly those in the compliant subgroup, used acute care services less frequently than the control group. Acute-care usage declined as less expensive health care resources were accessed. With nurse coaching, telemonitored patients initiated more phone contact with primary health care providers. Based on telemonitor data, nurses coaching responses to variations in daily health status resulted in patients having an increased desire for contact with their clinician. Bowles and colleagues [15] similarly indicated that in-home nursing visits (5 vs. 4.2), longer home care episodes (54 vs. 35 days) and nurse contacts were significantly increased for tele-homecare versus usual care. Conversely, Shea and Chamoff [16] found an inverse relationship between frequent remote patient-nurse communication and patient use of telemonitored data in their daily lives as they self-managed. Changes in signs/ symptoms were detectable through home-telemonitoring surveillance. Our study observed a favorable trend in the telemonitored compliant group of oncology Appalachians toward more positive outcomes than the control group. In respect to the study’s patient outcomes, functional status and QOL for lung cancer telemonitored patients throughout 60 days following acute-care or outpatient services were steadily enhanced in contrast to the control group. Responses to telemonitoring in our study were consistent with the overall reports of systematic reviews [14, 33]. Similar findings were also reported in other studies using comparable home telehealth programs among diverse patient populations [12, 16, 31, 32]. Use of telemonitored data conveying sufficient and continued information can improve patients’ self-management behaviors in community sites [9, 29, 30]. On the other hand, it could potentially prompt an overestimate of the actual need for health care, in turn, predisposing more frequent provider contact/ ER visits and jeopardizing the desired balance of cost-effectiveness. It is possible that use of additional health care resources may not have been initiated without patient self-management [9]. Daily monitoring of physiological parameters with nurse telephone coaching follow-up during the often unstable first two weeks post-hospitalization or post-clinical visit can be a cost-effective nursing approach in terms of patient survival [9, 14].

Our study suggests that QOL for Appalachians with lung cancer in mountainous, rural settings can be improved, although overall costs might be higher. Higher costs must be interpreted with caution. Unlike previous studies [31–33], evidence of cost-saving by using the home-telemonitoring alone was restricted in its significance in this pilot. Study data, in fact, showed lower costs for the control group which could be explained by earlier deaths in this group of advanced cancer patients. Nevertheless, longer survival is more expensive for the health care system, and best practices for provision of health care services in remote areas remain unclear. Further study with long-term cost analyses is needed. Moreover, according to a systematic review by Polisena et al. [12], a clear majority of home telehealth studies reported having reduced health care costs when telehealth is substituted for more expensive home care services, although two out of the 22 studies did not (one had increased costs and the other had no difference compared to usual care). Our study examined costs, clinical outcomes related to a specific disease, usual care as a comparator, and patients’ QOL that could be used to calculate QALYs in future studies. Because this was a pilot study, we did not estimate costs at a population level, include varied subject populations, or include marginal and sensitivity analyses. With a longer follow-up, health service costs could be further offset by decreased use of expensive hospital resources. On the other hand, if non-telemonitored patients die sooner than telemonitored patients, the conundrum of death actually lowering costs in the “usual care” group would continue. It remains to be decided if higher costs and higher QOL for patients with lung cancer may still indicate a reasonable tradeoff.

Receptiveness to telehealth care was strong and was perceived as beneficial by lung cancer patients and their families in this sample. Although conveying wireless data in mountainous remote surroundings was challenging, the feasibility of technology use and the at-home intervention protocol was confirmed and highly accepted. From the patients’ perspective, day-to-day monitoring was easy to use and empowered them with needed information to become more actively involved in managing their disease. Additionally, our data establishing satisfaction with telemonitoring was consistent with improved functional status and QOL. Although the positive patient outcomes found in our study are not evident in all other telemonitoring study results [14, 17, 31], the high patient satisfaction findings are consistent with three studies using different types of telehealth technologies (telephone-based and internet/ email alert-based) for cancer interventions [28, 30, 34].

This pilot study had several limitations. Clearly, in this particular sample with a high critical illness status, we experienced unanticipated difficulty and challenges with enrollment, recruitment, and retention, resulting in a relatively small sample size and a large proportion of missing data over time. The intention-to-treat strategy was employed in dealing with missing data for statistical analysis, and as a pilot study, statistical power was not a major concern. However, high attrition would still have impacted the detection and interpretation of the intervention’s appropriateness and effectiveness, similar to other studies with different patient characteristics [28, 35]. Our study findings indicated little strength in representing long-term clinical outcomes and intervention effects on cost. The long-term effect was not generalizable with two weeks of intervention surveillance and a two-month study follow-up. In addition, the cost calculation was conducted using primarily the cost of acute care services, including inpatient and scheduled cancer treatment/ checkups. Other intervention-associated expenditures or indirect costs, such as substituting telemonitor costs, for staff labor and travel time to patient homes were not considered, so a true cost-benefit analysis was not possible. Nevertheless, this pilot study builds a foundation for a future, larger RCT protocols to validate patient and clinical outcomes, particularly if an enrollment and retention research plan and strategies specific to this Appalachian patient group could be developed. In addition, further in-depth cost analysis is needed, including calculation of health care providers’ time and effort.

## Conclusion

To our knowledge, this pilot is the first to implement a home-based telemonitoring surveillance program for patients diagnosed with lung cancer in mountainous, rural Appalachian settings. Our study findings suggest that telemonitored data can be used to guide care from a distance and educate these patients to develop self-management skills when living with lung cancer. Although conducting research in critically ill patients residing in remote Appalachian areas is culturally, as well as practically challenging, this home-telemonitoring surveillance study establishes a feasible and acceptable protocol to enhance traditional practice. Telemonitoring-based patient education offers a potentially promising option to encourage patients to develop self-managed individualized care at home, maintain their health status, and ultimately improve patient-centered outcomes. Future research is warranted and essential to confirm the positive findings and cost-effectiveness with statistical significance and identify barriers related to improving patient self-management for optimal clinical outcomes. As a final point, research associated with self-management should continue to focus on the disadvantaged and underserved populations living in remote territories to minimize health disparities.

## Figures and Tables

**Table 1:** Patient Demographic and Clinical Characteristics.

Characteristics	Total (N=47)	Telemonitored (N=26)	Control (N=21)

Age (years; *M* ± SD; Range)	63 ± 9.9 (45–83)	63 ± 8.9 (49–80)	63 ± 11.3 (45–83)

Gender: Male (*N*; %)	26 (55)	14 (54)	12 (57)

Race: Caucasian (*N*; %)	46 (98)	26 (100)	20 (95)

Education level (%)			

No degree / Elementary	12 (25.6)	7 (27)	5 (23.8)
High school	24 (51.0)	14 (54)	10 (47.6)
College and above	11 (23.4)	5 (19)	6 (28.6)

Marital status (*N*; %)	32 (68.1)	18 (69.2)	14 (66.7)

Smoking History (*N*; %)			

Never	2 (4.3)	1 (3.8)	1 (4.8)
Past smoker	32 (68.0)	16 (61.5)	16 (76.2)
Current smoker	13 (27.7)	9 (34.6)	4 (19.0)

Household income (*N*; %)			

$0~$25,000	19 (40.4)	10 (38.5)	9 (42.9)
$25,000~$50,000	17 (36.2)	9 (34.5)	8 (38.1)
> $50,000	11 (23.4)	7 (27.0)	4 (19.0)

Health coverage			

Medicare	24 (51.0)	12 (46.2)	12 (57.1)
Medicaid	6 (19.1)	5 (19.2)	4 (19.0)

BMI (Ib/In^2^; *M* ± *SD*)	26.7 ± 6.5	25.7 ± 4.9	27.9 ± 7.9

Overweight/obesity (*N*; %)	29 (61.7)	14 (53.8)	15 (71.4)

Comorbidity (CCI^a^ index; *M* ± SD)	4.7 ± 3.5	4 ± 3.1	5.4 ± 3.7

Score >= 3 (*N*; %)	30 (63.8)	17 (65.4)	13 (61.9)

Type of lung cancer (*N*; %)			

NSCLC^b^	43 (91.5)	23 (88.5)	10 (95.2)
SCLC	4 (8.5)	3 (11.5)	1 (4.8)

Advanced cancer (Stages IIIB/IV/ extensive lesion; *N*; %)	31 (66.0)	16 (61.5)	15 (71.4)

Time since cancer diagnosis (< 1 year; *N*; %)	37 (77.8)	21 (84.0)	15 (71.4)

Cancer treatment (N; %)			

Completed	10 (21.3)	8 (30.8)	2 (9.5)
Ongoing	37 (78.7)	18 (69.2)	19 (90.5)

Hospitalization within the previous year^c^	36 (77.0)	23 (88.5)	13 (61.9)

Notes.

a CCI= Charlson Comorbidity Index;

b NSCLC= Non-small cell lung cancer & SCLC= small cell lung cancer;

c Group difference was found at previous hospitalization (*X^2^*= 4.57, *p* = .03). All other characteristics had no statistical difference between groups.

**Table 2:** Physiological Parameters at Hospital Discharge/Clinic Visit and 14- Days Post-discharge/Visit (Intervention Group Only).

Time/Measurement	Temp (F)	Pulse (bpm)	Sys^a^ BP (mmHg)	Dias^b^ BP (mmHg)	Wt^c^ (lbs)	O_2_Sat^d^ (%)
At discharge	97.1	89	118	69	167	94
(Range)	(95.1–99.0)	(60–111)	(83–144)	(51–89)	(103–230)	(88–99)
14 days Post-discharge	97	90	126	73	162	94
(Range)	(95.2–99.2)	(59–118)	(97–170)	(56–96)	(105–217)	(78–99)

Notes.

a Systolic blood pressure;

b Diastolic blood pressure;

c Body weight;

d Oxygen saturation with/ without oxygen. All physiological parameters were shown in average (available *N*= 15) and had no significant difference at baseline compared to 14-days post- hospital discharge/clinic visit (*p* >.05).

**Table 3:** Aggregate Costs^a^ over Two-Month Study Time Periods.

	Control Group			Telemonitored Group		
	Obs	Mean	St. Err.	Obs	Mean	St. Err.
**Pharmaceutical**	21	$8,114.24	$1,375.06	26	$8,372.63	$1,352.07
**Lab/Diagnostic test**	21	$23,944.97	$3,938.07	26	$27,532.52	$4,922.00
**Surgery**	21	$21,839.56	$5,906.03	26	$22,157.79	$4,428.55
**Room**	21	$19,004.20	$3,245.75	26	$18,917.36	$2,695.96
**Other**	21	$3,163.25	$1,247.16	26	$5,197.06	$3,132.32
**Total**	21	$76,066.23	$12,472.50	26	$82,177.37	$10,754.49

Note.

a This table implicitly imputes zero when costs are unobserved (*p* = .711).

Obs: observed data; St. Err.: Standard error.
